# Sequential egocentric navigation and reliance on landmarks in Williams syndrome and typical development

**DOI:** 10.3389/fpsyg.2015.00216

**Published:** 2015-02-25

**Authors:** Hannah J. Broadbent, Emily K. Farran, Andrew Tolmie

**Affiliations:** ^1^Psychology and Human Development, University College London, Institute of EducationLondon, UK; ^2^Centre for Brain and Cognitive Development, School of Psychology, Birkbeck, University of LondonLondon, UK

**Keywords:** Williams syndrome (WS), navigation, visuospatial cognition, egocentric, landmarks

## Abstract

Visuospatial difficulties in Williams syndrome (WS) are well documented. Recently, research has shown that spatial difficulties in WS extend to large-scale space, particularly in coding space using an allocentric frame of reference. Typically developing (TD) children and adults predominantly rely on the use of a sequential egocentric strategy to navigate a large-scale route (retracing a sequence of left–right body turns). The aim of this study was to examine whether individuals with WS are able to employ a sequential egocentric strategy to guide learning and the retracing of a route. Forty-eight TD children, aged 5, 7, and 9 years and 18 participants with WS were examined on their ability to learn and retrace routes in two (6-turn) virtual environment mazes (with and without landmarks). The ability to successfully retrace a route following the removal of landmarks (use of sequential egocentric coding) was also examined. Although in line with TD 5-year-olds when learning a route with landmarks, individuals with WS showed significantly greater detriment when these landmarks were removed, relative to all TD groups. Moreover, the WS group made significantly more errors than all TD groups when learning a route that never contained landmarks. On a perceptual view-matching task, results revealed a high level of performance across groups, indicative of an ability to use this visual information to potentially aid navigation. These findings suggest that individuals with WS rely on landmarks to a greater extent than TD children, both for learning a route and for retracing a recently learned route. TD children, but not individuals with WS, were able to fall back on the use of a sequential egocentric strategy to navigate when landmarks were not present. Only TD children therefore coded sequential route information simultaneously with landmark information. The results are discussed in relation to known atypical cortical development and perceptual-matching abilities in WS.

## INTRODUCTION

Williams syndrome (WS) is a neurodevelopmental disorder arising from a deletion of around 27 genes on chromosome 7q11.23 (Koehler et al., 2014). Characteristic of WS is an uneven cognitive profile of relative strengths and weaknesses, with poor visuospatial skills relative to verbal abilities frequently reported (e.g., Jarrold et al., 1998). The use of an allocentric spatial frame of reference to guide navigation (coding spatial relationships between objects external to the self) is particularly problematic for individuals with WS as evidenced on both on small- (Nardini et al., 2008; Bernardino et al., 2013) and large-scale tasks (Farran et al., 2010; Broadbent et al., 2014). However, it is currently unclear whether such spatial difficulties in WS are also evident in the use of an egocentric frame of reference (coding spatial relationships between the self and environmental objects) during large-scale navigation.

In the real world, navigation usually requires the ability to retrace a route from one location to another, along familiar or previously learnt paths, only occasionally necessitating knowledge of short-cuts or an understanding of the allocentric relationships between locations in a given environment. Indeed, some research has argued that even when taking novel short-cuts, a simple egocentric landmark navigation strategy is predominantly employed, rather than complex allocentric spatial referencing (Foo et al., 2005, 2007). That said, the extent to which landmarks are used to facilitate route-learning, and the specific function that they serve, has been a matter of some debate. Some authors have reported the importance of environmental landmarks as navigational aids (e.g., Jansen-Osmann, 2002), while others have found little benefit of the presence of landmarks, particularly when other strategies, such as recalling a sequence of left–right turns, are readily available to the navigator (e.g., Tlauka and Wilson, 1994).

The ability to use landmarks to guide performance on spatial tasks develops throughout early childhood (e.g., Newcombe and Huttenlocher, 2000; Sutton, 2006). Although a range of ages have been suggested as to when the ability to use different landmarks emerges, this likely relates to variability in task demands, the size of environmental space, and the role that landmarks are given during learning (Waller and Lippa, 2007). In relation to the development of large-scale spatial knowledge, spatial representations become progressively more complex throughout childhood, with landmark knowledge conjectured to precede sequential route knowledge and global or configurational mental representations, respectively (Siegel and White, 1975; Cousins et al., 1983). This developmental acquisition of spatial knowledge is considered to be mediated by the emergence of low-level cognitive abilities, with landmark knowledge associated with the development of recognition-in-context memory, and route knowledge with the emergence of paired-associative learning (Allen and Ondracek, 1995). Others have concluded that these age-related changes are associated with a hierarchical development of spatial coding, rather than a qualitative shift from one stage to the next (e.g., Newcombe and Huttenlocher, 2000). This is also in line with different developmental trajectories for egocentric and allocentric spatial frames of reference as seen in both small- (Nardini et al., 2006) and large-scale tasks (Bullens et al., 2010), with older children and adults more able to successfully switch to using an allocentric frame of reference when the task requires a more global mental representation of space.

It can therefore be inferred that younger children will rely on more basic landmark-based spatial strategies by which to navigate, that do not comprise of allocentric (spatial relational) information. Cohen and Schuepfer (1980) showed children aged 7 and 11 years and adults a series of slides along a route that contained landmarks. Although all groups required the same amount of time to learn the route, when shown the slides of the maze without landmarks, 7-year-olds made more incorrect turn choices than 11-year-olds, who in turn made more errors than adults. The youngest children also recalled fewer landmarks that were integral to route orientation (i.e., positioned at a correct or incorrect turn). These findings were replicated in the same age groups using continuous navigation through virtual environments (VEs; Jansen-Osmann and Wiedenbauer, 2004). However, Jansen-Osmann and Fuchs (2006) argued that the findings of the two aforementioned studies actually indicate that children and adults use landmark information in a qualitatively similar way to enhance way-finding, and that developmental differences in the use of landmarks are only seen in relation to other aspects of spatial cognition. That is, although 11-year-olds and adults were able to learn a route in fewer trials than 7-year-olds (both with and without landmarks), way-finding performance in all groups benefited equally from the presence of landmarks. The findings did, however, show a developmental difference in landmark knowledge and orientation behavior. Similarly, when asked to retrace a route, children aged 6 and 12 years of age were found to benefit equally from being advised to take notice of landmarks near the route; although only the older children were able to benefit from being told to pay attention to distant landmarks (Cornell et al., 1989). As such, children and adults are seen to use the information provided by (proximal) landmarks to a similar extent to adults for way-finding behavior, but a developmental difference is seen in the way in which landmarks are used for other aspects of large-scale spatial cognition, with age-related differences in the use of landmarks dependent on the task demands and type of landmark available.

On navigation tasks designed to elicit spontaneous navigation strategies, typically developing (TD) children and adults predominantly rely on a sequential egocentric strategy, recounting the sequence of left–right body turns to retrace a route (Iglói et al., 2009; Bullens et al., 2010). Given the importance of egocentric spatial coding and use of landmarks as navigational guides, it is therefore essential to address the way in which individuals with WS are able to navigate from one location to another along familiar routes, and which aspects of the environment are the most useful in aiding successful way-finding.

On a comparable task to that by Bullens et al. (2010), individuals with WS were found to exhibit atypical navigational performance, indicating that the use of more simple sequential egocentric strategies may be problematic for this group (Broadbent et al., 2014). These findings suggest instead that individuals with WS may rely on less efficient navigational methods. One possibility, explored in the present study, is that individuals with WS use a technique that involves searching for familiar visual scenes in order to find a target location.

Individuals with WS are able to successfully learn a route both in virtual and real environments, albeit often at a slower rate than TD children of comparable non-verbal ability (Farran et al., 2010, 2012a,b). However, to date, this has only been examined in environments in which landmarks have been present. As such, research investigating the extent to which individuals with WS rely upon the presence of landmarks to guide learning and the retracing of a route would provide further insight into the specific navigation strategies employed by this group. More specifically, an examination into the ability to use a sequential egocentric strategy when explicitly required to do so (for example, when landmarks are removed and an individual must rely on their memory of the sequence of left–right body turns) would be a useful tool to further identify specific deficits in large-scale spatial cognition in WS.

To date, there has been a paucity of research examining the role of landmarks in navigation in WS. In a 6-turn VE, individuals with WS were able to successfully learn a route using landmarks as cues to aid way-finding, in line with TD children aged 6–8 years (Farran et al., 2012a). The study also found that individuals in the WS group with higher non-verbal ability were able to differentiate between junction and path landmarks. This was shown by superior memory for junction over path landmarks, and was therefore indicative of an ability to understand the usefulness of landmarks at junctions. As a result, the authors concluded that although individuals with WS are able to form cognitive representations of landmarks, important landmark knowledge that can be used to enhance way-finding may only occur with increased maturity of non-verbal ability.

These findings suggest that environmental landmarks may play an important role in the development of spatial knowledge in WS, as seen in typical development. Furthermore, previous findings suggest that individuals with WS may well use visual scenes within an environment to navigate, including in situations where TD children are able to apply alternative spatial coding strategies (Broadbent et al., 2014). The extent to which individuals with WS would rely on the presence of landmarks both for learning and retracing a route compared to TD children, however, remains equivocal.

Allocentric spatial coding is associated with preferential activity in the right hippocampal region (e.g., Burgess et al., 2002; Hartley et al., 2003), with ‘egocentric’ processing associated with activation in the fronto-parietal networks along the dorsal stream (e.g., Seubert et al., 2008; Weniger et al., 2009). However, memory for the sequence of body turns associated with specific choice points through a route (a sequential egocentric strategy) may involve other neural networks than those associated with independent egocentric responses. Indeed, during the use of a sequential egocentric strategy the left hippocampus is preferentially activated (Iglói et al., 2010). Related, sequential memory is associated with activation in the hippocampus, particularly during the acquisition of spatial sequences (Rolls and Kesner, 2006). These findings are in line with cortical activation during sequential route-based navigation in mice (Rondi-Reig et al., 2006). In light of these findings regarding the neural basis of the use of different spatial frames of reference and navigational strategies, alongside findings of atypical brain development in WS, particularly in the hippocampus (Meyer-Lindenberg et al., 2005), it stands to reason that individuals with WS would exhibit specific difficulties on tasks requiring accurate processing of both allocentric and sequential egocentric spatial information. However, it remains unclear as to the strategies that individuals with WS typically use to complete way-finding tasks, and indeed, the underlying neural mechanisms and cortical structures that are involved.

The aim of the present study was to therefore examine the extent to which individuals with WS rely on the presence of landmarks both when learning a route, and when retracing a route once landmarks are removed following learning, compared to TD children of comparable verbal and non-verbal ability. In essence, are individuals with WS able to apply the use of a sequential egocentric strategy (recalling the sequence of left–right body turns), which is independent of the use of landmarks, to retrace a route when visual properties of the environment (i.e., landmarks) are no longer, or have never been, available?

Given the above mentioned discussion alongside findings of atypical strategy use during spontaneous navigation tasks in WS (Broadbent et al., 2014), it was inferred that individuals with WS would have difficulties in developing a sequential egocentric representation of a route that could be used when landmarks were removed. TD children and adults are able to reiterate the sequence of body turns through a route to reach a target location (Iglói et al., 2009; Bullens et al., 2010). However, atypical brain development in cortical regions that subserve spatial coding strategies such as allocentric and sequential egocentric representations in WS imply that individuals with this disorder will rely heavily on landmarks, and to a greater extent even than TD children when learning a route. Impairments in the use of a sequential egocentric strategy would also be reflected in a difficulty learning a route that does not contain any visual landmark cues, and would therefore be indicative of a complete reliance on landmarks to guide way-finding.

With regards to the nature of difficulties in the use of a sequential egocentric strategy in WS, a further aim of this study was to examine the types of errors made by individuals with WS compared to TD children when learning routes in environments with and without landmarks. That is, do individuals with WS present with a similar pattern of errors in environments with and without landmarks as seen in TD children?

## MATERIALS AND METHODS

### PARTICIPANTS

Fifty-three TD children were recruited from two London, UK primary schools, and separated into three age groups; 5-, 7-, and 9-year-olds. None of the TD children were known to have any developmental disorders (as acknowledged by parents and teachers) and all participants had normal or corrected-to-normal vision. Twenty-one individuals with WS were recruited from the records of the Williams Syndrome Foundation, UK. All participants with WS had received a positive diagnosis of WS, based on a “fluorescence *in situ* hybridization” (FISH) test for deleted Elastin gene on the long arm of chromosome 7, as well as phenotypic diagnosis from a clinician. Written informed consent was obtained from the parents of all participants. Signed individual consent was additionally collected from participants with WS over 12 years of age. Ethical approval for the study was received through the Institute of Education London ethics committee.

All TD participants were tested in a quiet room within their schools, whilst WS participants were tested either at their home or in a testing room at the Institute of Education, London. Five participants from the TD groups; 5 years (*N* = 3), 7 years (*N* = 1), and 9 years (*N* = 1), and three participants from the WS group had difficulties with the tasks or did not complete all measures. Data for these participants were subsequently excluded from the analyses. Therefore, data were analyzed from 48 TD children [5 years: (*N* = 16, mean age (years; months) = 5.09, SD = 0.09, range = 5.03–6.01), 7 years: (*N* = 16, mean age = 7.08, SD = 0.02, range = 7.05–8.00), 9 years: (*N* = 16, mean age = 9.07, SD = 0.03, range = 9.03–10.00)], and 18 participants with WS (Mean age = 21.09, SD = 4.07, range = 16.01–32.01). Verbal and Non-verbal abilities were assessed using the British Picture Vocabulary Scale-III (BPVS-III; Dunn et al., 2009) and the Ravens Coloured Progressive Matrices (RCPM; Raven et al., 2003), respectively.

### VIRTUAL ENVIRONMENT

**FIGURE 1:**
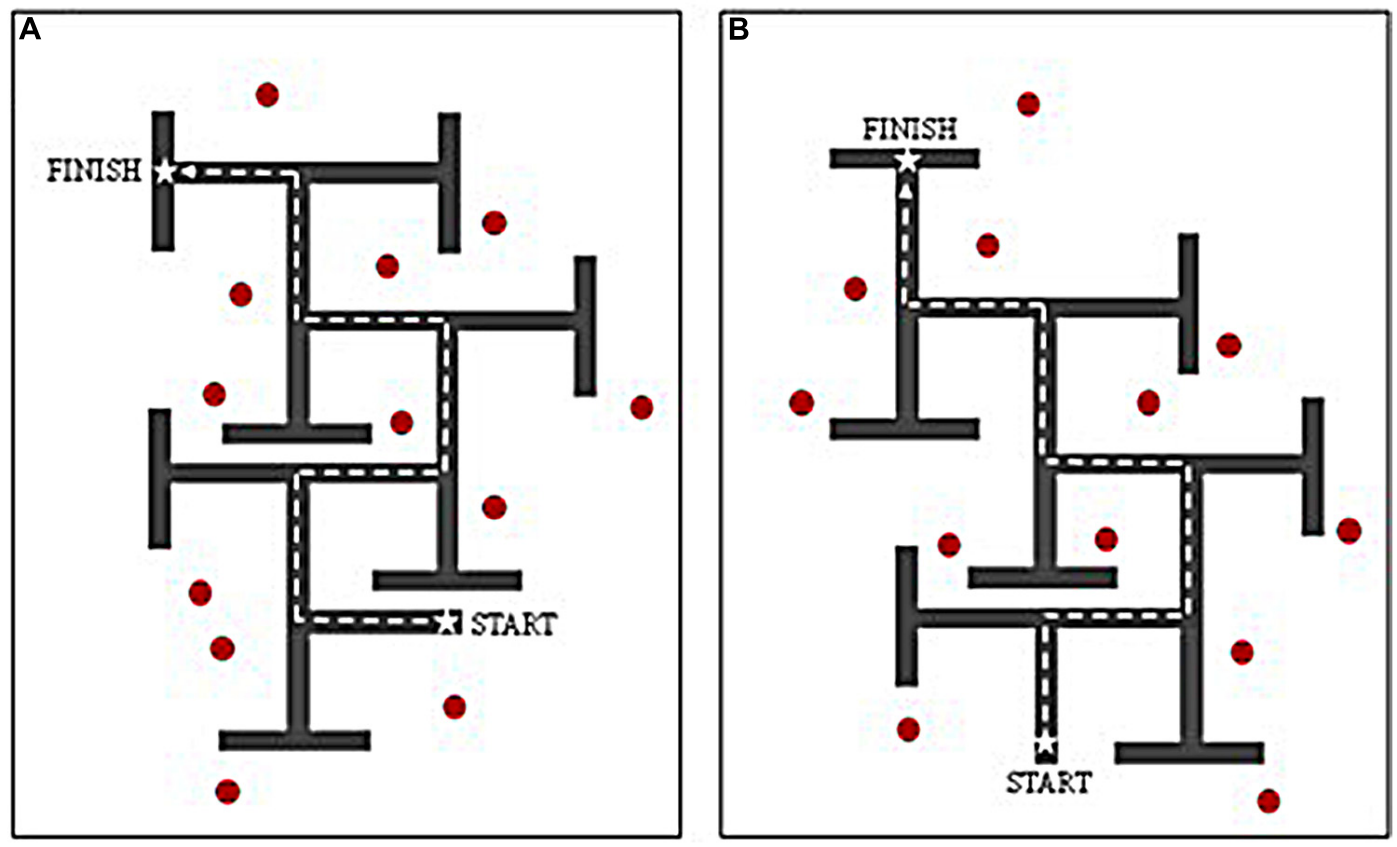
**Birds-eye-view of virtual environment routes **(A,B)** used for counterbalancing.** Dashed line denotes correct route through maze. Red markers denote location of distal landmarks when route used for ‘landmark’ condition.

The interactive VE was developed using The Vizard Development Edition (version 3.0) software program, and presented on a 17” laptop screen. The VE task presented participants with a 6-turn maze layout within which individuals were able to navigate using the arrow keys on the keyboard. Each path was of equal length, and each decision point consisted of a single left–right T-junction. Incorrect turns were concealed using T-junctions at the end of each dead-end path, so that they did not appear visually different from correct turns when viewed from the decision point. Each participant completed a ‘landmark (LM)’ and ‘no-landmarks (NLM)’ condition, the order of which was counterbalanced across participants in all groups. Two maze designs (layouts A and B) were employed; these contained identical path lengths, structure, and wall height, but with different sequences of left–right turns (see **Figure 1**). To control for any differences across maze designs, half of the participants in each group received layout A as the LM condition and layout B as the NLM condition, whilst the other half received layout B as the LM condition and layout A as the NLM condition. For consistency, the same set of landmarks was used for each LM condition, regardless of maze design (A or B), with comparable configurations.

To distinguish between conditions and notify participants they were learning a different route, the LM condition always consisted of red-brick walls, whereas the NLM condition maze always contained gray-brick walls, regardless of whether layout A or B was used. This was kept consistent for all participants so that LM scenes presented in a ‘visual-matching’ task presented following the LM trials (detailed below), all contained red brick walls.

There were no time restrictions for completion of any of the tasks, and navigation speed within the VEs was set to a consistent pace that initial pilot testing suggested was optimal for participants in each group to easily traverse the route without disorientation or boredom.

### DESIGN AND PROCEDURE

All participants completed the BPVS-III and RCPM tasks before any other measures. Participants were then presented with either the LM or NLM condition VE maze (counterbalanced across participants in each group). A ‘visual-matching’ and ‘landmark naming’ task always immediately followed the LM condition for all participants.

#### ‘Landmark’ condition

In the LM condition, participants were asked to navigate from the starting position to find a “hidden exit” at the end of the maze (either layout A or B). Surrounding the maze were 12 distal landmarks (two distinct trees, two distinct lampposts, a playground, a cityscape, a high-rise building, a house, a traffic light, a red tower, a shop, and a block of flats), at least two of which were visible from each path. During the learning phase, participants were first shown the correct route by following a grass path. On reaching the exit, a celebratory trumpet sound was played and the program window automatically closed. The participant was then returned to the starting position, without the grass path, for the first learning trial. Here, participants were required to walk the route from memory. An error was counted if the participant traveled more than half way down an incorrect path. During the learning trials, the participant had to navigate through the correct route to the exit without error on two consecutive trials to reach learning criterion and move onto the test trial. Each participant was given a maximum of seven learning trials to reach criterion, unless zero errors were made on trial 7, then eight trials were given. As this was to check whether learning had occurred within seven trials, if errors were made on trial 8, data from this trial were not included in the analysis. This learning criterion was selected based on previous findings in TD and WS that showed if participants had not learnt a route after this number of trials then they will continue to have difficulty in successfully learning the route (Broadbent et al., 2014).

Once the participant had learnt the route successfully (reached criterion) in the landmark condition, they were returned to the starting position of the maze, but this time with all landmarks removed. Participants were then asked to retrace the correct route to the exit. This single test trial was used to examine the extent to which participants continued to rely on the use of landmarks for successful navigation following learning, or whether they were able to use sequential egocentric coding to retrace the route.

#### ‘No landmarks’ condition

As a control condition, to examine whether participants were able navigate through a maze that did not have any landmarks to begin with, participants were also asked to learn a route through the NLM. Similar to the LM condition, participants were first shown the correct route by following a grass path to the hidden exit. Participants then entered the learning phase, without the grass path and were asked to navigate the correct route, without error. In line with LM condition learning trials, participants had to navigate the correct route with no errors on two consecutive trials (maximum seven trials, unless zero errors on trial 7, then eight trials) to meet criterion.

#### ‘Visual-matching’ task

Immediately following the LM condition, participants were presented with a visual-matching task, to examine the ability to remember the correct visual scenes from the maze. Participants were shown a series of images from the viewpoint of each of the six junctions of the maze that they had learned. For each trial, two images (one correct and one incorrect) were presented adjacent to each other on the computer screen. Participants were asked to select which of the two scenes they had actually viewed when walking through the maze. Incorrect scenes consisted of either an incorrect configuration (an incorrect spatial layout, but containing the correct landmarks), or included incorrect landmarks from different parts of the maze (but in the same configuration as the correctly presented scene). Each correct and incorrect scene pair was presented twice throughout the task (12 trials), with each image appearing once on the left- and once on the right-hand-side of the screen.

#### ‘Landmark naming’ task

To examine whether participants were able to easily recognize and name each landmark, and therefore, to assess the saliency of each environmental marker as a potential navigational guide, a landmark naming task was used. Here, each of the twelve landmarks from the landmark-maze was presented individually on the screen and the participant was asked to name the object. Items were scored as correct if the correct name, a commonly used alternative, or synonym was given.

## RESULTS

### STATISTICAL ANALYSES

Data were examined for deviations from normality using Kolmogorov–Smirnov tests (*p* < 0.05). Given the robustness of parametric tests to variations of normality (Field, 2009), parametric tests were conducted throughout. Non-parametric equivalents were also conducted in cases where data for half of the groups were not normally distributed, with comparable results. Therefore only the results of parametric analyses are presented. One exception is for ‘number of learning trials’ in the NLM condition. Here, data were not normally distributed for any group (Kolmogorov–Smirnov, *p* < 0.01 for all). In addition, all WS participants (and the majority of TD participants) failed to meet learning criterion for the NLM, and thus reached the maximum trial limit. For this reason, non-parametric analyses were conducted on this data and analyzed separately from LM data.

### VERBAL AND NON-VERBAL ABILITIES

To examine differences across groups on BPVS-III and RCPM scores, one-way analyses of variance (ANOVA) were conducted for both measures, with group (four levels: 5, 7, 9 years, and WS) as a between-subjects factor. Results demonstrated an uneven cognitive profile in WS, characteristic of the disorder (Jarrold et al., 1998; Martens et al., 2008), with non-verbal abilities significantly below TD 9-year-olds, and at a level no different from TD 5- and 7-year-olds, compared to relatively higher verbal abilities, significantly greater than TD 5- and 7-year-olds, but not significantly different from TD 9-year-olds (**Table 1**).

**Table 1:** Mean (SD) raw scores on BPVS-III and RCPM for each group.

	Group				ANOVA		*Post hoc*^a^
	WS (*N* = 18)	5 years (*N* = 16)	7 years (*N* = 16)	9 years (*N* = 16)	*F (df)*	*p*	
BPVS^b^	128.39 (15.38)	76.19 (6.78)	91.56 (12.85)	120.06 (11.26)	67.39 (3,65)	<0.001	5 < 7 < 9 = WS
RCPM^c^	20.83 (6.21)	17.75 (2.54)	21.56 (3.97)	28.06 (4.11)	15.05 (3,65)	<0.001	WS = 5 = 7 < 9

*^a^Tukey-corrected *post hoc* tests, ‘=’ refers to no significant difference at 0.05 level, and ‘<’ denotes ‘significantly less than’ (*p* < 0.01).*

*^b^BPVS-III, British Picture Vocabulary Scale-III raw scores.*

*^c^RCPM, Ravens Coloured Progressive Matrices (RCPM) raw scores.*

### LEARNING PHASE

To examine route-learning abilities in both the ‘with landmarks’ (LM) maze condition and ‘no-landmarks’ (NLM) maze condition across groups, Mean number of learning trials taken to reach criterion (two consecutive trials without error) was calculated for each maze condition in each group. As a more sensitive measure of route-learning ability, the cumulative number of errors made across all learning trials for each maze condition was also analyzed. Descriptive statistics for these two dependent variables are displayed in **Table 2**.

**Table 2:** Group means (SD) for measures of performance on learning phase in landmark (LM) and no-landmark (NLM) mazes.

		Group			
		WS (*N* = 18)	5 years (*N* = 16)	7 years (*N* = 16)	9 years (*N* = 16)
LM	Number of learning trials *(including two criterion trials)*	5.17 (1.89)	4.25 (1.84)	3.50 (1.16)	3.25 (1.44)
	Number of errors	5.06 (4.35)	3.19 (3.47)	1.44 (1.26)	2.00 (2.19)
NLM	Number of learning trials *(including two criterion trials)*	7.00 (0.00)	5.75 (1.77)	5.94 (1.48)	5.50 (2.16)
	Number of errors	15.94 (3.72)	9.06 (6.65)	7.94 (5.98)	7.81 (6.48)

#### Number of learning trials to reach criterion

Results of a one-way ANOVA (with Tukey-corrected pairwise comparisons) for number of learning trials on the LM maze (including the two correct criterion trials), showed a significant effect of group, *F*(3,65) = 4.85, *p* = 0.004, η^2^ = 0.19. This was due to the WS group requiring a significantly greater number of trials to learn the route than TD 7-year-olds and 9-year-olds; *p =* 0.020 and *p =* 0.006, respectively. No significant differences were found between WS and TD 5-year-olds (*p* = 0.360), or between any TD groups (*p* > 0.05 for all).

For the NLM, results of a Kruskal–Wallis test yielded a significant effect of group on number of learning trials, *H*(3) = 8.43, *p* = 0.038. *Post hoc* Mann–Whitney tests showed that this was due to the WS group requiring a significantly greater number of trials than TD 5-year-olds (*U* = 90.00, *z* = –2.81, *p* = 0.005, *r* = –0.48), TD 7-year-olds (*U* = 81.00, *z* = –3.08, *p* = 0.002, *r* = –0.53), and TD 9-year-olds (*U* = 90.00, *z* = –2.32, *p* = 0.021, *r* = –0.39). No significant differences were found between any TD groups (*p* > 0.05 for all).

#### Number of errors across learning trials

To examine the effects of counterbalancing on mean number of errors, separate mixed ANOVAs, with between-participant factor of groups (four levels: 5, 7, 9, and WS) and within-participant factors of ‘maze’ (two levels: LM and NLM) included either a between-participant factor of ‘maze-order’ (two levels; order 1: LM before NLM; order 2: NLM before LM)’ or ‘route-order’ (two levels: A first; B first).’ Given that no significant main effects were found for either ‘maze-order’ (*F* < 1) or ‘route-order’ (*F* < 1), nor were there any significant interactions with either variable (*p* > 0.05 for all) it was concluded that there were no order effects, and so these variables were not included in subsequent analyses that examined error types.

**FIGURE 2:**
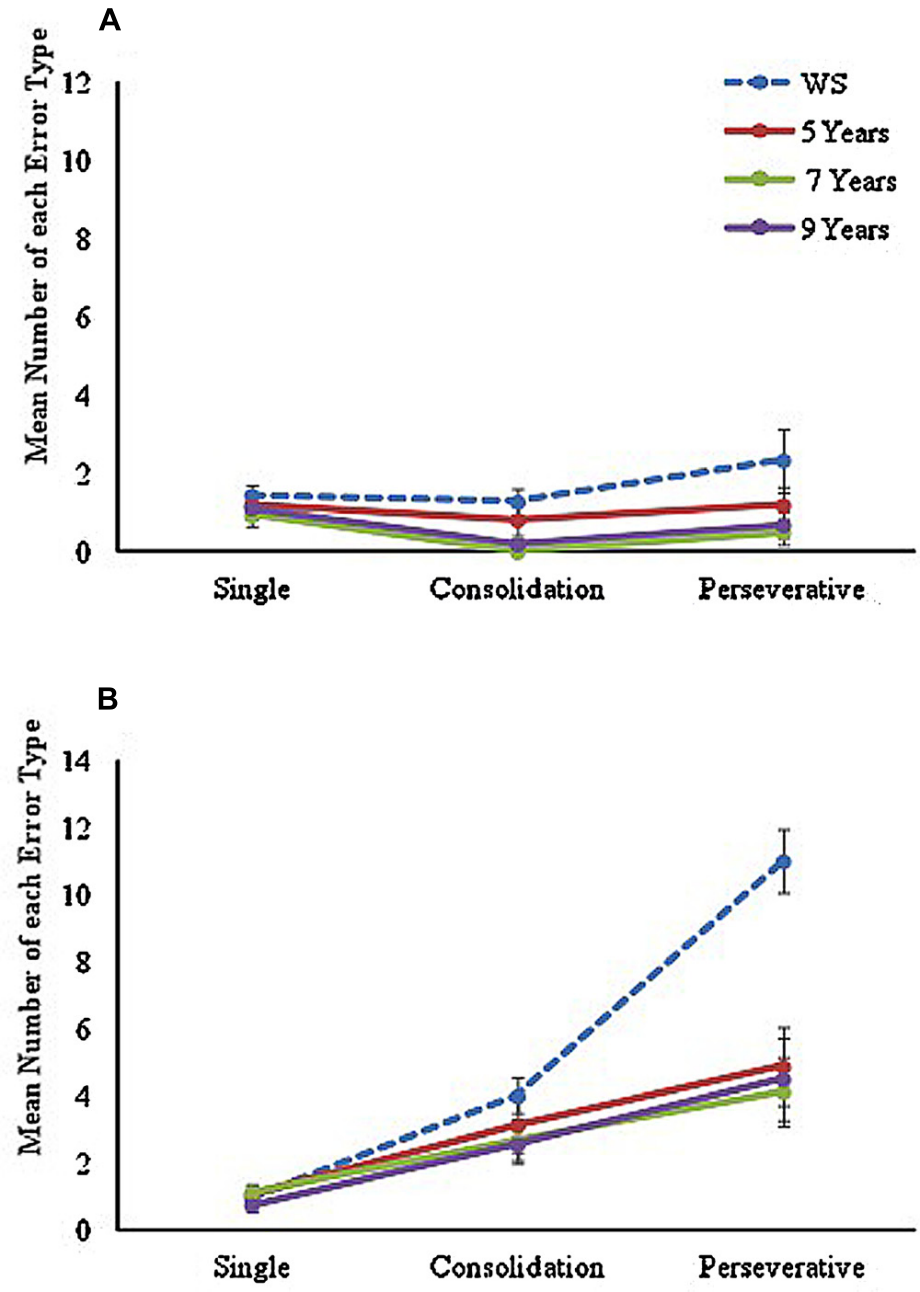
**Mean number of each error type made across learning trials on **(A)** landmark maze and **(B)** no-landmark maze conditions for each group**.

To examine the number and type of errors made during the learning trials for each maze condition, errors were separated into three categories, in line with the method used by Farran et al. (2012a), and included as a within-participants factor in the analyses. Errors were coded as (a) ‘single errors’ (an error that occurred only once at a specific junction across all learning trials), (b) ‘consolidation errors’ (errors that occurred at the same junction on more than one learning trial, but not on consecutive trials), or (c) ‘perseveration errors’ (errors that occurred at the same junction on two or more consecutive learning trials). Mean number of each type of error made during the LM and NLM conditions are displayed in **Figure 2**.

A mixed ANOVA with between-participants factor of ‘group’ (four levels: 5, 7, 9, and WS) and two within-participant factors of ‘maze’ (two levels: landmark and no-landmark) and ‘error type’ (three levels: single, consolidation, and perseverative) was conducted. A significant main effect of group [*F*(3,62) = 10.77, *p* < 0.001, $\eta_{p}^{2}$ = 0.34] was found, with Tukey-corrected *post hoc* comparisons showing this was due to the WS group making a significantly greater number of errors than all TD groups (*p* < 0.05 for all). No significant difference was found between any TD groups (*p* > 0.05 for all). A significant main effect of maze [*F*(1,62) = 89.93, *p* < 0.001, $\eta_{p}^{2}$ = 0.59] was also found with significantly poorer performance on the NLM (*p* < 0.001). Results also found a significant main effect of Error Type, *F*(1.43,88.59) = 44.72, *p* < 0.001, $\eta_{p}^{2}$ = 0.42, with pairwise comparisons indicating a significant difference across all error types (*p* < 0.001 for all). The analysis also revealed a near-significant group by maze, *F*(3,62) = 2.67, *p* = 0.055, $\eta_{p}^{2}$ = 0.11, and significant maze by error-type, *F*(1.87,115.94) = 46.22, *p* < 0.001, $\eta_{p}^{2}$ = 0.43, and error-type by group, *F*(4.29,88.59) = 6.99, *p* < 0.001, $\eta_{p}^{2}$ = 0.25 interactions. A significant 3-way maze by error-type by group interaction was also found, *F*(5.21,107.74) = 3.98, *p* = 0.002, $\eta_{p}^{2}$ = 0.16, showing that there was a different pattern of errors seen between the two mazes, that differed across groups. Given the significant main effects, significant interactions, and difference in number of trials to reach criterion, each maze was analyzed separately.

The analysis of types of errors made in the LM condition learning trials showed a significant main effect of group, *F*(3,62) = 4.56, *p* = 0.006, $\eta_{p}^{2}$ = 0.18, with Tukey-corrected *post hoc* tests showing that the WS group made significantly more errors than the TD 7 (*p* = 0.006) and 9 (*p* = 0.028) year-olds, but were not significantly different from TD 5-year-olds (*p* = 0.306). A significant effect of error type was also found, *F*(1.45,89.82) = 4.25, *p* = 0.028, $\eta_{p}^{2}$ = 0.06. Pairwise comparisons demonstrated that this was due to significantly fewer consolidation errors than single or perseverative errors (*p* = 0.003 for both). However, no significant error-type by group interaction was found, *F* < 1, showing that the pattern of errors observed in the LM did not differ across groups.

Results for the NLM condition also showed a significant main effect of group, *F*(3,62) = 7.87, *p* < 0.001, $\eta_{p}^{2}$ = 0.28. Here however, Tukey-corrected pairwise comparisons showed that the WS group made a significantly greater number of errors than all TD groups on this maze (*p* < 0.01 for all). This was different to performance in the LM condition, in which the WS group performed in line with TD 5-year-olds, and thus explains the marginal maze by group interaction. No significant differences were found across TD groups (*p* > 0.05 for all). An analysis of error type in the NLM condition learning trials, also found a significant main effect of error type, *F*(1.75,106.41) = 55.18, *p* < 0.001, $\eta_{p}^{2}$ = 0.47, due to a significant difference across all error types (*p* < 0.001 for all). In addition, a significant error type by group interaction was found, *F*(5.15,106.41) = 6.64, *p* < 0.001, $\eta_{p}^{2}$ = 0.24.

To examine this interaction further, error type was examined separately across groups. Results of a one-way ANOVA revealed a significant difference only in mean number of perseverative errors across groups, *F*(3,65) = 9.49, *p* < 0.001. Tukey-corrected pairwise comparisons showed that this was due to a significantly greater number of perseverative errors made by the WS group than any TD groups (*p* ≤ 0.001 for all). No significant differences across TD groups were found (*p* > 0.05 for all).

### ‘LANDMARKS-REMOVED’ TEST TRIAL

**FIGURE 3:**
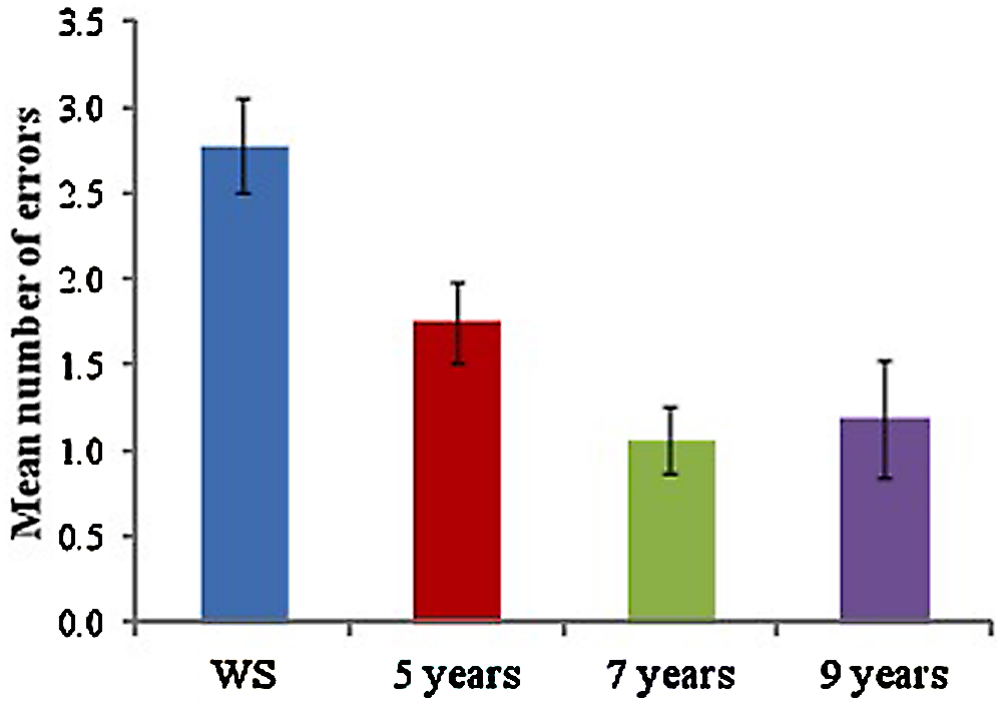
**Mean number of errors made during ‘landmarks removed’ test trial for each group**.

As a measure of the ability to use a sequential egocentric strategy following learning in the landmark-condition, participants were asked to immediately complete the route one final time, with all landmarks removed. Mean number of errors made during the test trial was calculated for each group (see **Figure 3**). A one-way ANOVA to examine the mean number of errors made across groups, yielded a significant difference across groups, *F*(3,65) = 8.89, *p* < 0.001, with Tukey-corrected pairwise comparisons showing that the WS group made a significantly greater number of errors than all TD groups (5 years, *p* = 0.038, 7 years, *p* < 0.001, 9 years, *p* < 0.001). No significant differences were found across TD groups (*p* > 0.05 for all).

### ‘VISUAL-MATCHING’ TASK

Incorrect scenes on the visual-matching task were separated into those with an incorrect configuration (but with correct landmarks for that junction) and those that included incorrect landmarks (but in the correct configuration for that junction). Mean number of errors (incorrect scene choices made, out of 12 trials) and Mean number correct were calculated for each group.

Although significantly below ceiling (using one-sample *t*-tests with Mean number correct), all groups performed well on this task, with few errors made [5 years: *M* = 9.44 (1.67), *t*(15) = –6.13, *p* < 0.001; 7 years: *M* = 9.63 (1.67), *t*(15) = –5.69, *p* < 0.001; 10 years: *M* = 9.75 (2.69), *t*(15) = -3.34, *p* = 0.004; WS: *M* = 9.22 (1.89), *t*(17) = –6.22, *p* < 0.001].

**FIGURE 4:**
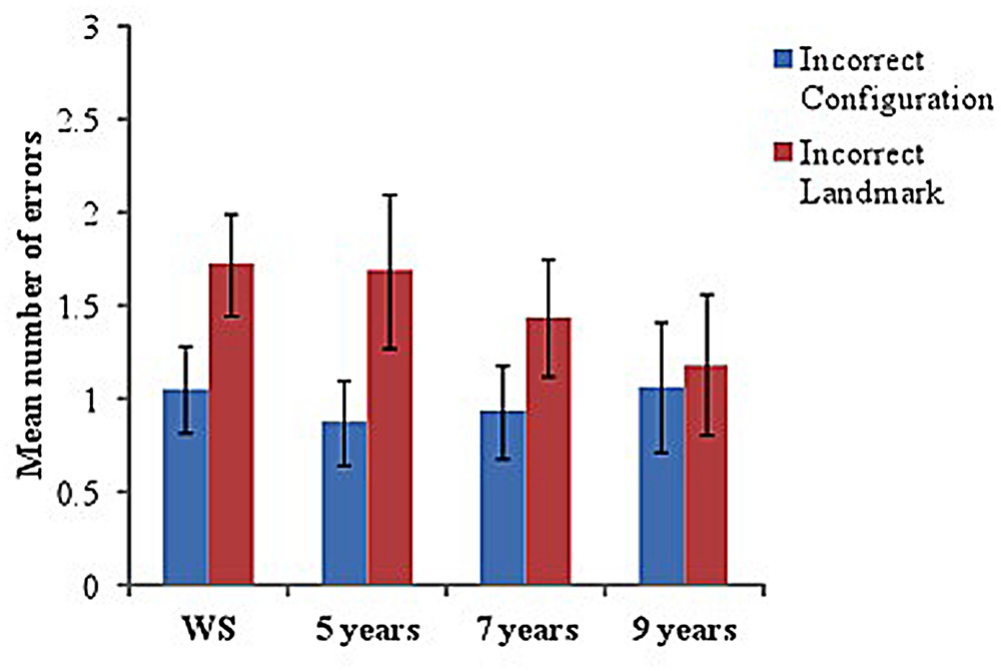
**Mean number of errors (incorrect scenes chosen) on ‘visual-matching’ task in each group.** Incorrect scenes are separated into those that included an ‘incorrect configuration’ and those with an ‘incorrect landmark.’

To examine errors across groups, a 2-way ANOVA with a between-participant factor of group (four levels: WS, 5, 7, and 9 years) and within-participant factor of ‘scene type’ (two levels: incorrect configuration and incorrect landmark) was conducted. No significant differences were identified across groups, *F* < 1, indicating proficient ability to visually match correct scenes from a recently learned maze in all groups (see **Figure 4** for Mean errors in each group). However, a significant effect of scene type was found, *F*(1,62) = 8.17, *p* = 0.006, $\eta_{p}^{2}$ = 0.12, with pairwise comparisons indicating that participants made significantly more errors when the incorrect visual scene contained an incorrect landmark compared to an incorrect configuration (*p* = 0.006).

### ‘LANDMARK NAMING’ TASK

To examine the saliency of each environmental feature used in the landmark condition maze, the number of correct or appropriate labels given for each of the 12 landmarks was calculated for each group. All landmarks were easily named, with a high number of correct labels given by each group [Mean (SD)]; WS: 11.83 (0.38), 5 years: 11.50 (0.82), 7 years: 11.69 (0.60), 9 years: 11.94 (0.25).

## DISCUSSION

The present study examined the extent to which individuals with WS rely on the presence of landmarks both for learning and for retracing a route, compared to TD children aged 5–9 years. On learning a six-turn route in a VE maze with 12 distal landmarks, individuals with WS performed in line with TD 5-year-olds, although required a significantly greater number of trials and made more errors than TD children aged 7 and 9 years. This finding is in line with that of previous route-learning studies in WS, showing route-learning performance at a level expected based on non-verbal reasoning ability (Farran et al., 2010, 2012a). On learning trials in a maze without landmarks (NLM), however, individuals with WS presented with a significantly higher level of impairment than all TD groups, with none of the participants in the WS group successfully able to learn the route. As such, although all groups demonstrated substantially poorer performance on learning a maze without landmarks compared to one with landmarks, the negative impact on learning without visual cues for navigation was seen to a greater extent in individuals with WS than all TD groups. This difficulty in navigating without the presence of visual cues was further substantiated when landmarks were removed following learning in the LM condition. Here, even having successfully learnt the route with landmarks to a level comparable with TD 5-year-olds, individuals with WS made reliably more errors than all TD groups once these were removed, demonstrating a greater reliance on the presence of visual markers to guide learning and subsequent repetition of a series of decision points along a route.

No significant differences were found in performance across the three TD groups on measures of route-learning in either maze condition, nor on number of errors made following the removal of landmarks. This absence of a developmental difference on these tasks is indicative of, not only a high level of route-learning ability by 5 years of age, but also the capacity to fall back on the use of a sequential egocentric navigation strategy when required from at least 5 years. This is, in part, counter to earlier findings of age-related changes in a reliance on landmarks to make correct turns at decision points along a newly learnt route (e.g., Cohen and Schuepfer, 1980; Jansen-Osmann and Wiedenbauer, 2004). The use of proximal landmarks in the above-mentioned studies, compared to the use of distal landmarks in the present study may underlie these different findings, particularly given that distal (global) landmarks may have allowed participants to develop a single, integrated representation of the route (Ruddle et al., 2011), resulting in less reliance on landmarks, even in the youngest group. Alternatively, given that the age range in the present study did not extend as high as in previous studies, it is possible that the addition of an older TD group here may have yielded improvements with age. That said, the present results are in line with findings that young children use landmark information to enhance way-finding to the same extent as older children and adults (Jansen-Osmann and Fuchs, 2006). In addition, the present findings alongside previous findings (Bullens et al., 2010; Broadbent et al., 2014) suggest that the use of sequential egocentric coding is a viable navigation strategy that can be used by children as young as 5 years of age. This is akin to the spontaneous navigation strategy predominantly employed by typical adults on similar route-learning tasks (Iglói et al., 2009).

The high level of ability to successfully retrace a route observed in TD children following the removal of landmarks begs the question of whether participants in these groups were simply remembering a verbal sequence of left–right turns to complete the route, and were thus unfazed by the change in environmental appearance. There are three arguments to counter this. First, children aged 5 years have difficulty in the use and representation of ‘left/right’ spatial terms (Landau and Hoffman, 2005) and so successful execution of this strategy would have been particularly problematic in this group. Second, errors (albeit very few) were observed following landmark removal in all TD groups, indicating the role of landmarks at some level in supporting the development of spatial knowledge in these groups. Third, all TD groups performed highly on the visual-matching task, demonstrating that they had attended to and encoded this information for use in way-finding, a process that would not be necessary had they solely relied upon verbally labeling the left–right sequence. It can be inferred therefore that during learning in TD children, but not in individuals with WS, a sequential egocentric representation of the temporal order of bodily turns was developed simultaneously to the paired-associative learning of directional responses to specific landmarks.

The use of a sequential egocentric strategy in which the temporal sequence of body turns is encoded, requires cognitive demands corresponding to those required for episodic or procedural memory (Packard and McGaugh, 1996; Iglói et al., 2009), and is associated with activation in the left hippocampus (Iglói et al., 2010). Given the known cortical atrophy associated with WS that includes the hippocampus (Meyer-Lindenberg et al., 2005), it stands to reason that spatial encoding that is typically supported by these neural networks would be impaired in this disorder. Indeed, structural and functional abnormalities in the hippocampal region in WS are likely associated with not only impairments in the use of an allocentric spatial frame of reference in WS (Broadbent et al., 2014), but also with difficulties in the use of a sequential egocentric representation, as identified in the present study.

Examining the categorization of errors provided further insight into the nature of impairments in the use of a sequential egocentric strategy in WS. In particular, the high number of perseverative errors in the WS group, compared to other error types and to performance by all TD groups reflected a difficulty in WS in learning a 6-turn sequence of turns. This difference may simply reflect the natural outcome of the scoring criteria when a high total number of errors are made (e.g., during the NLM). At a behavioral level, perseverative errors have been previously noted in WS during navigation tasks (Mandolesi et al., 2009; Farran et al., 2012a). This is indicative of difficulties inhibiting previous incorrect responses and is supported by findings that individuals with WS fail to engage cortical and subcortical structures in the frontostriatal regions that mediate response inhibition (Mobbs et al., 2007). In the current study, however, a significantly greater number of perseverative errors in WS compared to TD groups was not identified during learning in a maze with landmarks. Alternatively, the presence of visual cues in the LM condition may have supported the formation of cognitive representations of landmarks to guide way-finding in the WS group, leading to fewer perseverative errors. In contrast to the present findings, Farran et al. (2012a) found a high number of perseverative errors in WS. The use of proximal landmarks in the Farran et al. (2012a) study and the use of distal landmarks in the present study may provide the crucial comparison for future research.

The substantial impairments in WS on tasks that required sequential egocentric coding suggest that individuals with this disorder instead rely on the presence of landmarks to navigate, and to a greater extent even than TD children of comparable non-verbal ability. Successful use of landmarks for way-finding on a previously traversed route requires storage of visual information at decision points, even if the sequential order of these choice points are not encoded. As such, if individuals with WS rely on the use of a visual-matching strategy to navigate, it is reasonable to surmise that they would score highly on tests of scene recognition. This hypothesis was supported in the current study, showing that participants in this group were successful at identifying correct visual scenes from the maze with landmarks. This is in line with findings from an individual with bilateral hippocampal damage who presented with spatial impairments in allocentric and context-dependent episodic memory, but was able to recognize scenes from a VE environment, suggesting that visual pattern-matching is not associated with the hippocampus (Spiers et al., 2001). Previous research in WS has also shown that some perceptual abilities may be relatively unimpaired. For instance, although individuals with WS are typically impaired on block construction tasks (e.g., Bellugi et al., 1988), performance on perceptual components of similar tasks are at the level of mental-age matched controls (Rondan et al., 2003; Deruelle et al., 2006).

On the visual-matching task, the finding that incorrect-landmark scenes were more difficult to determine than incorrect-configuration scenes was not anticipated; an outcome that was particularly unexpected in WS, given the known difficulties with coding configurational information in this group (e.g., Nardini et al., 2008; Bernardino et al., 2013). However, this distinction may have been related to differences in the ability to detect changes in fine and coarse visual information. For instance, in the incorrect-landmark scenes, the general outline of the scene would have been similar to the correct layout, making it more difficult to disambiguate between correct and incorrect choices than with changes to coarse information, as seen in incorrect-configuration scenes. However, it is difficult to make robust conclusions from this task, particularly given that in some scenes more landmarks were visible than in others, meaning that scenes were not matched for level of difficulty. An alternative method for future studies using such visual-matching tasks should therefore include an incorrect-landmark and incorrect-configuration version of each scene for comparison. That said, it is important to note the high level of accuracy in all TD groups and the WS group on both scene-types, indicating that all groups were able to use the visual information from the environment to guide way-finding by some means.

## CONCLUSION

Individuals with WS demonstrate a reliance on visual landmarks for route-learning and way-finding, to a greater extent than TD children of comparable non-verbal ability. All participants with WS failed to learn a route that did not contain landmarks, which required the development of a representation of the temporal sequence of body turns. When learning a route with landmarks, TD children, but not individuals with WS, were able to simultaneously develop a sequential egocentric representation of the route to aid way-finding in situations such as when landmarks were removed. Individuals with WS instead likely relied on a visual-matching strategy by which to navigate, which is susceptible to errors following changes to the physical presentation of the environment (i.e., the removal of landmarks). Impairments in memory processes involved in episodic spatial events such as remembering the sequence of bodily rotations through a route is in line with atypical development in associated cortical regions in WS. These findings provide insight, not only into the impairments in WS in large-scale spatial cognition, but into the strategies that may be commonly employed by individuals with this disorder to support way-finding, when typical strategies and egocentric and allocentric spatial frames of reference are not available to them.

## Conflict of Interest Statement

The authors declare that the research was conducted in the absence of any commercial or financial relationships that could be construed as a potential conflict of interest.
